# *Stahlhut, Björn/Lammert, Martin (Hrsg.)*: Gesamtstaatliche Sicherheitsvorsorge – gerüstet für den Ernstfall!?, 200 S., BWV, Berlin 2022.

**DOI:** 10.1007/s42520-023-00496-5

**Published:** 2023-05-25

**Authors:** Jonas Franken, Christian Reuter

**Affiliations:** Wissenschaft und Technik für Frieden und Sicherheit (PEASEC), Technische Universität Darmstadt, Darmstadt, Deutschland

Das Sicherheitsumfeld Deutschlands und Europas ist im dynamischen Wandel begriffen. Als Antwort auf die daraus erwachsenen Herausforderungen wurde in den vergangenen Jahren wiederholt eingefordert, staatliche Ansätze müssten „interministeriell“, „ressortübergreifend“, „vernetzt“, „gesamtstaatlich“, oder gar „resilient“ sein. Der Sammelband „Gesamtstaatliche Sicherheitsvorsorge – gerüstet für den Ernstfall!?“ reiht sich somit in eine langwierige Debatte ein. Im Titel stellt er immerhin die zentrale Frage: Ist die Sicherheitsarchitektur Deutschlands in der Praxis fähig, den vielfältigen Bedrohungen der Gegenwart und Zukunft zu begegnen?

Akademische Veröffentlichungen zur Sicherheitspolitik leiden häufig unter eben dieser volatilen Dynamik, sollen sie doch den Spagat zwischen zwei kaum zu vereinbaren Polen leisten: Einerseits müssen sie möglichst aktuell Prozesse einordnen und faktenbasierte Lösungsvorschläge für identifizierte Missstände erarbeiten. Andererseits muss dabei die akademische Gründlichkeit gewahrt werden, insbesondere in den langwierigen Veröffentlichungsprozessen. Dem Sammelwerk der Herausgeber Björn Stahlhut und Martin Lammert gelingt der Spagat allerdings nur zum Teil.

Die Entstehungszeit der Beiträge lässt sich auf das Jahr 2021 eingrenzen. Entsprechend prägnant wird der Fokus auf die damals diskursdominierende COVID-19-Pandemie gelegt. So finden sich unter den 18 Beiträgen gleich vier mit einem gesundheitspolitischen Fokus (Stahlhut, Annika Vergin, Johannes Richert, Isabell Halletz), was auch in der Position des Mitherausgebers Stahlhut, der sich selbst als „Mittler und Übersetzer zwischen Gesundheitspolitik und Sicherheitspolitik“ beschreibt, begründet liegen mag. Diese vier Beiträge sind allesamt spannend zu lesen und enthalten für die Nachbereitung der pandemischen Lage wichtige Einblicke. Insbesondere Halletz legt unter Verweis auf zahlreiche Statistiken die Notwendigkeit einer zukunftsfähigen Altenpflege nachvollziehbar dar. Die sonst interessanten Nischenbeiträge von Richert zur Verfasstheit des Roten Kreuzes und Vergin zum *Human Performance Enhancement* fallen dagegen leicht ab, weil ihre Kontribution zur überspannenden Frage des Werks nicht ersichtlich wird.

Die Entstehungszeit führt zudem dazu, dass mehrere heute sicherheitspolitisch prägende Ernstfälle nicht diskutiert werden konnten. Allen voran der russische Angriffskrieg gegen die Ukraine, der eine zumindest proklamierte „Zeitenwende“ der Sicherheitspolitik eingeläutet hat, fehlt in den Betrachtungen. Obwohl dieser zeitgeschichtliche Fakt keineswegs den Herausgebern vorzuwerfen ist, leidet darunter doch die Relevanz des Gesamtwerks für Sicherheitsprobleme der Gegenwart. Die Beiträge über die von der Zeitenwende besonders betroffenen außen- und verteidigungspolitischen Handlungsfelder der Landes- und Bündnisverteidigung enthalten dennoch aufschlussreiche Elemente.

Patrick Sensburgs vorausschauender Appell für eine stärkere Reservearbeit fußt argumentativ bereits auf die Rückbesinnung Deutschlands und der NATO auf Landes- und Bündnisverteidigung seit dem russischen Angriff auf die Ukraine 2014. Auch Siemtje Möller nimmt Bezug auf die Annexion der Krim und leitet nachvollziehbare Schlussfolgerungen für die maritime Sicherheit ab. Allerding hat sich dort nach den Sabotagen der Ostsee-Pipelines Nord Stream im September 2022 mit dem Schutz maritimer Kritischer Infrastrukturen bereits ein neues Betätigungsfeld für die Marine aufgetan, das mittlerweile höhere Wichtigkeit einnimmt als die anekdotisch beschriebene Präsenz im Indo-Pazifik. Der Kommandeur des Cyber- und Informationsraums der Bundeswehr Thomas Daum beleuchtet in seinem Beitrag die Rolle der Streitkräfte in Zeiten der digitalen Transformation und geht dabei in einer Tiefe, die die Mehrheit der weiteren Beiträge vermissen lässt, auf die tatsächlichen Kooperationen staatlicher Stellen im Sinne des ressortübergreifenden Ansatzes ein.

Bei den innenpolitischen Beiträgen treten die Kritikpunkte deutlicher zutage. Zunächst variieren die Abstraktionsniveaus und die wissenschaftliche Qualität der Beiträge stark. Das obere Ende der Qualitätsskala machen logisch argumentierte und ordentlich referenzierte Beiträge aus (Sabina Wölkner, André Algermissen, Thomas Müller-Färber, Lammert). Die Mitte der Skala bilden die praxisnahen Berichte (Irene Mihalic, Janosch Dahmen, Verena Schäffer, Julia Höller), die in erster Linie für thematisch Interessiere lesenswert sein dürften. Der Band enthält allerdings auch mehrere Beiträge, die offenbar eines deutlich umfassenderen Lektorats bedurft hätten (Christian Sprengel, Burkard Dregger) oder recht einseitige Beiträge mit Wirtschaftsfokus, die mehr oder minder verhohlen Lobbypapiere für Wasserstoffenergie (Katherina Reiche) beziehungsweise die Arbeitgeber_innenseite (Wolfgang Steiger) darstellen. Aufgeladene Begriffe wie der deplatzierte Vorwurf einer „moralinsauren, planwirtschaftlichen Haltung mit Hang zu Verboten und staatlicher Gängelei“ (S. 172) werfen Fragen hinsichtlich der Autor_innenauswahl für das ökonomische Feld auf. Die „Streitschrift“ von Dregger fällt durch eine Argumentationsweise auf, die zumindest als einseitig, in der dargestellten Art gegebenenfalls als plump empfunden werden könnte. Darüber müssen sich auch die Herausgeber im Klaren gewesen sein, sahen sie sich genötigt, den teils an problematischen Stereotypen orientierten (S. 128) und entgegen der erwiesenen terroristischen Gefährdungslage übermäßig auf Linksextremist_innen fokussierten Beitrag als einzigen vorweg einordnen zu müssen (S. 126). Dreggers eingeforderte rabiate Grundrechtseingriffe wie elektronische Fußfesseln oder gar Präventivgewahrsam gegen Gefährder_innen lassen sich nicht als verfassungsrechtlich vertretbare Maßnahme erkennen.

Die Varianz der Beitragsqualität erschwert es Lesenden ungemein, einen kohärenten Argumentationsstrang zu identifizieren, der die aufgeworfene Frage umfassend beantworten könnte. Entsprechend zeigt der Sammelband auf beiden Seiten des bildlichen Spagats Limitationen: Unverschuldet ist er bereits nach weniger als einem Jahr seit Veröffentlichung teils nicht mehr aktuell und leidet bei manchen Beiträgen unter sichtbaren Schwächen in der akademisch-handwerklichen Umsetzung.

